# Role of melatonin as an SIRT1 enhancer in chronic obstructive pulmonary disease induced by cigarette smoke

**DOI:** 10.1111/jcmm.14816

**Published:** 2019-11-25

**Authors:** Na‐Rae Shin, Je‐Won Ko, Jong‐Choon Kim, Gunhyuk Park, Sung‐Hwan Kim, Min‐Seok Kim, Joong‐Sun Kim, In‐Sik Shin

**Affiliations:** ^1^ College of Veterinary Medicine (BK21 Plus Project Team) Chonnam National University Gwangju Korea; ^2^ Herbal Medicine Resources Research Center Korea Institute of Oriental Medicine Naju‐si Korea; ^3^ Jeonbuk Department of Inhalation Research Kore Institute of Toxicology Jeongeup Korea; ^4^ Human and Environmental Toxicology University of Science and Technology Daejeon Korea

**Keywords:** chronic obstructive pulmonary disease, cigarette smoke, melatonin, nuclear factor‐kappa B, SIRT1

## Abstract

**Background:**

Melatonin has various biological activities that improve the health of an individual. We evaluated the effects of melatonin on inflammatory response in chronic obstructive pulmonary disease (COPD), focusing on the regulation of SIRT1 expression.

**Methods:**

To investigate the effect of melatonin, we used cigarette smoke (CS)‐induced COPD mouse model and CS condensate (CSC)‐stimulated J774 macrophage cells.

**Results:**

CSC‐stimulated J774 macrophages exhibited increased p65 acetylation with a reduction in SIRT1 expression. However, melatonin induced the enhancement of SIRT1 expression, which eventually decreased p65 acetylation in CSC‐stimulated J774 cells. Melatonin‐treated mice exhibited an enhancement in SIRT1 expression with the reduction in p65 acetylation, which decreased the level of inflammatory mediators induced by CS. Additionally, SIRT1 inhibitor treatment increased the level of inflammatory mediators, which was accompanied by an increase in p65 acetylation. However, cotreatment with melatonin and an SIRT1 inhibitor reduced the level of inflammatory mediators compared with that by treatment with the SIRT1 inhibitor alone, which was accompanied by elevation in SIRT1 expression and reduction in p65 acetylation.

**Conclusions:**

Overall, the results indicated that melatonin has therapeutic effects against COPD, owing to its property to enhance SIRT1 expression.

## 1 INTRODUCTION

Chronic obstructive pulmonary disease (COPD) is one of the major causes of mortality worldwide and is characterized by airway inflammation and irreversible airflow obstruction.^1^ An important risk factor of COPD is cigarette smoke (CS), which induces the production of various cytokines and chemokines in the airways and the recruitment of inflammatory cells to the lung tissue.^2^ SIRT1, a class III histone deacetylase, has been recognized as an important regulator of inflammatory response; a high level of SIRT1 leads to the suppression of inflammatory response.^3^ CS reduces SIRT1 expression in the respiratory tract and activates the NF‐κB‐dependent inflammatory responses.^4^

Melatonin (*N*‐acetyl‐5‐methoxytryptamine), a natural hormone, is released from the pineal gland in mammals.^5^ Melatonin has been reported to have anti‐inflammatory and anti‐oxidant effects in clinical and experimental studies.^5^ In particular, its anti‐inflammatory effect is suggested to occur via the inhibition of various signalling pathways such as NF‐κB pathways.^6^, ^7^ Thus, we explored the effects of melatonin against CS and lipopolysaccharide (LPS)‐exposed mouse and CS condensate (CSC)‐stimulated J774 macrophages by measuring the inflammatory mediator levels and evaluating SIRT1 signalling.

## 2 MATERIALS AND METHODS

### 2.1 Experimental animal model

C57BL/6N male mice (6‐8 weeks) were obtained from Koatech, and the experiment was granted by the Institutional Animal Care and Use Committee of the Chonnam National University. The CS was produced from 3R4F research cigarettes (University of Kentucky) by a CS generator (Daehan Biolink). The animals were inhaled to CS for 1 h/d for 14 days and received an intranasal instillation of LPS (10 μg per mouse) on Days 4 and 11 while anesthetized. Melatonin (15 and 30 mg/kg, Sigma‐Aldrich) was intraperitoneally injected for 14 days, and SIRT1 inhibitor (Sirtinol, Sigma‐Aldrich) was intraperitoneally injected at 10 mg/kg on Day 3 and 10. To collect the bronchoalveolar lavage fluid (BALF), we performed a tracheostomy according to a previous study.^8^ The lungs were twice cold PBS (total volume 1.4 mL), and BLAF was centrifuged at 200 *g* for 5 minutes at 4°C. The supernatant was collected and used for ELISA, and the pellets were collected and used for cell analysis. Differential cell count was determined using the Diff‐Quik stain, and the level of tumour‐necrosis factor (TNF)‐α and interleukin (IL)‐6 was measured using ELISA kits (R&D System).

### 2.2 Immunoblotting and gelatin zymography

Immunoblotting was carried out according to a previous study.^8^ The following primary antibodies were used: anti‐SIRT1 (Abcam), anti‐acetylated NF‐κB (Abcam), anti‐NF‐κB (Abcam) and anti‐β‐actin (Abcam). Quantitative analysis of protein expression was performed using the IMT i‐Solution software (IMT i‐Solution Inc). Gelatin zymography was conducted according to a previous study.^9^

### 2.3 Histological studies

The sections were stained with haematoxylin and eosin (Sigma‐Aldrich). In addition, we performed immunohistochemistry (IHC) analysis according to a previous study.^8^ For IHC, analysis was deparaffinized, dehydrated and washed in PBS containing 0.05% tween 20. The slide was incubated to block nonspecific staining for 20 minutes at room temperature with goat serum. The slide was incubated with primary mouse anti‐rabbit SIRT1 antibody (Abcam) for 2 hours at room temperature. After incubation, the slides were incubated for 1 hour at room temperature with biotinylated secondary antibody and then incubated with an avidin‐biotin‐peroxidase complex (Vector Laboratories) for 1 hour at room temperature. Then the slides were washed and incubated with diaminobenzidine (DAB, Abcam) for an additional 5 minutes. The histological images were captured using IMTcamCCD5 (IMT i‐Solution Inc).

### 2.4 Cell culture

J774 macrophages (ATCC) were incubated in Roswell Park Memorial Institute 1640 medium supplemented with 10% foetal calf serum, antibiotics and HEPES. The cells were seeded in six‐well plates, treated with melatonin, and incubated with CSC for 24 hours. The cell culture supernatant was obtained to analyse the TNF‐α level. The cells were collected, and then Western blotting was performed. Immunocytochemistry (ICC) was performed as previously reported.^10^ Anti‐SIRT1 antibody (Abcam) was used as primary antibody. The slides were visualized using a confocal laser scanning microscope (LSM510m; Carl Zeiss).

### 2.5 Statistical analysis

The data are presented as mean ± standard deviation (SD). Statistical analyses were performed using GraphPad Prism 6. The values with *P* < .05 and <.01 were considered significant.

## 3 RESULTS

### 3.1 Effect of melatonin on SIRT1 expression in CSC‐stimulated J774 macrophages

**Figure 1:**
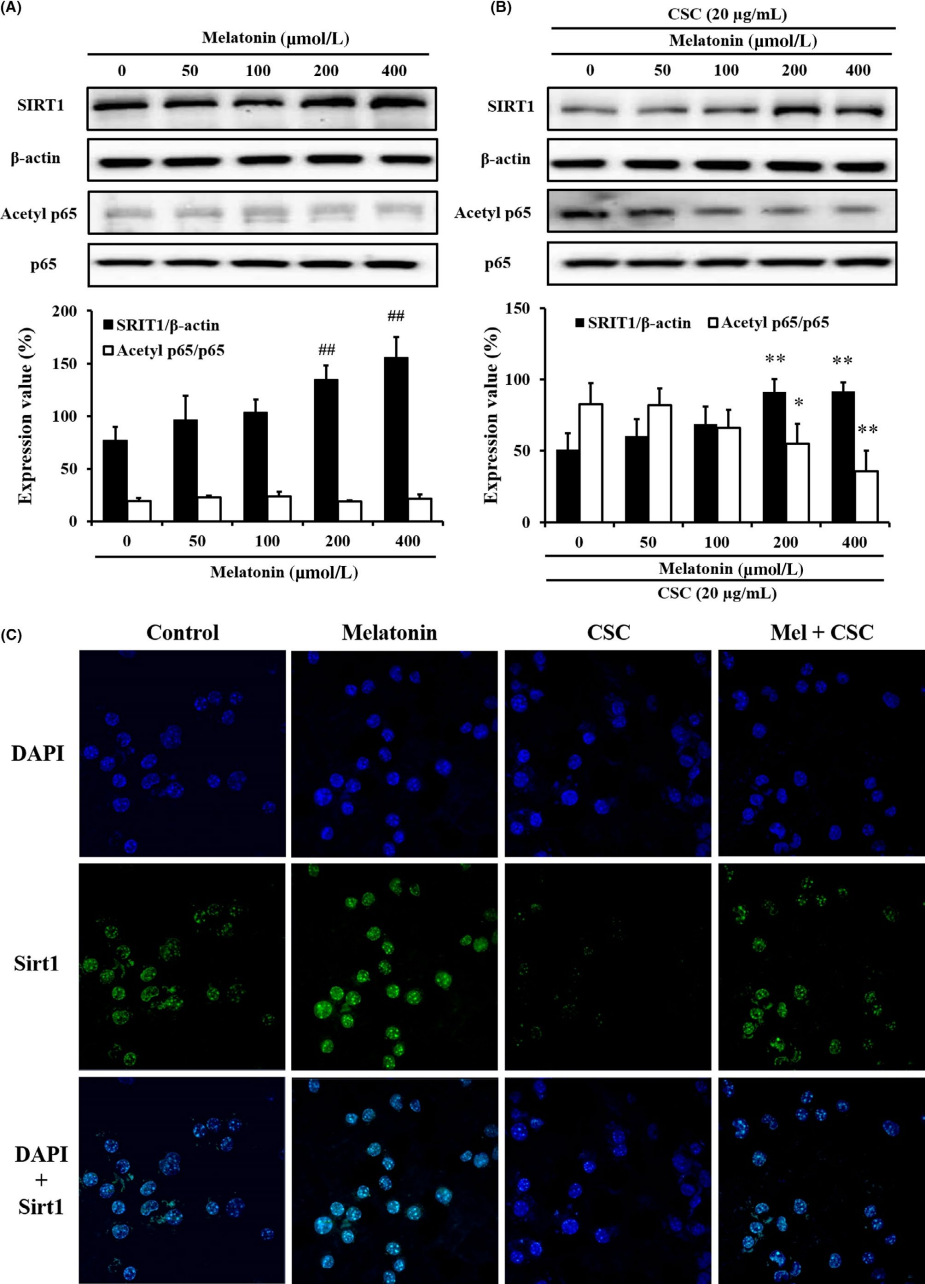
Melatonin enhanced SIRT1 expression in cigarette smoke condensate (CSC)‐treated J774 macrophages. A, SIRT1 expression in J774 macrophages; B, SIRT1 expression in CSC‐treated J774 macrophages; C, SIRT1 expression in immunocytochemical staining. ^##^ *P* < .01, compared with untreated J774 macrophages; **P < .01, compared with CSC‐treated J774 macrophages. The data are presented as mean ± standard deviation (SD). Statistical analyses were performed using analysis of variance, followed by a multiple comparison test with Dunnett adjustment using GraphPad Prism 6

The CSC‐treated J774 macrophages decreased SIRT1 expression and increased TNF‐α level (Figures S1 and S2). However, melatonin treatment decreased the TNF‐α level compared with that in CSC‐treated J774 macrophages. Melatonin treatment increased SIRT1 expression in non‐stimulated J774 macrophages in a concentration‐dependent manner (Figure 1A). Furthermore, melatonin treatment increased SIRT1 expression (Figure 1B,C) and decreased p65 acetylation in CSC‐stimulated J774 macrophages (Figure 1B).

### 3.2 Effect of melatonin on airway inflammation and SIRT1 expression in CS‐exposed mice

**Figure 2:**
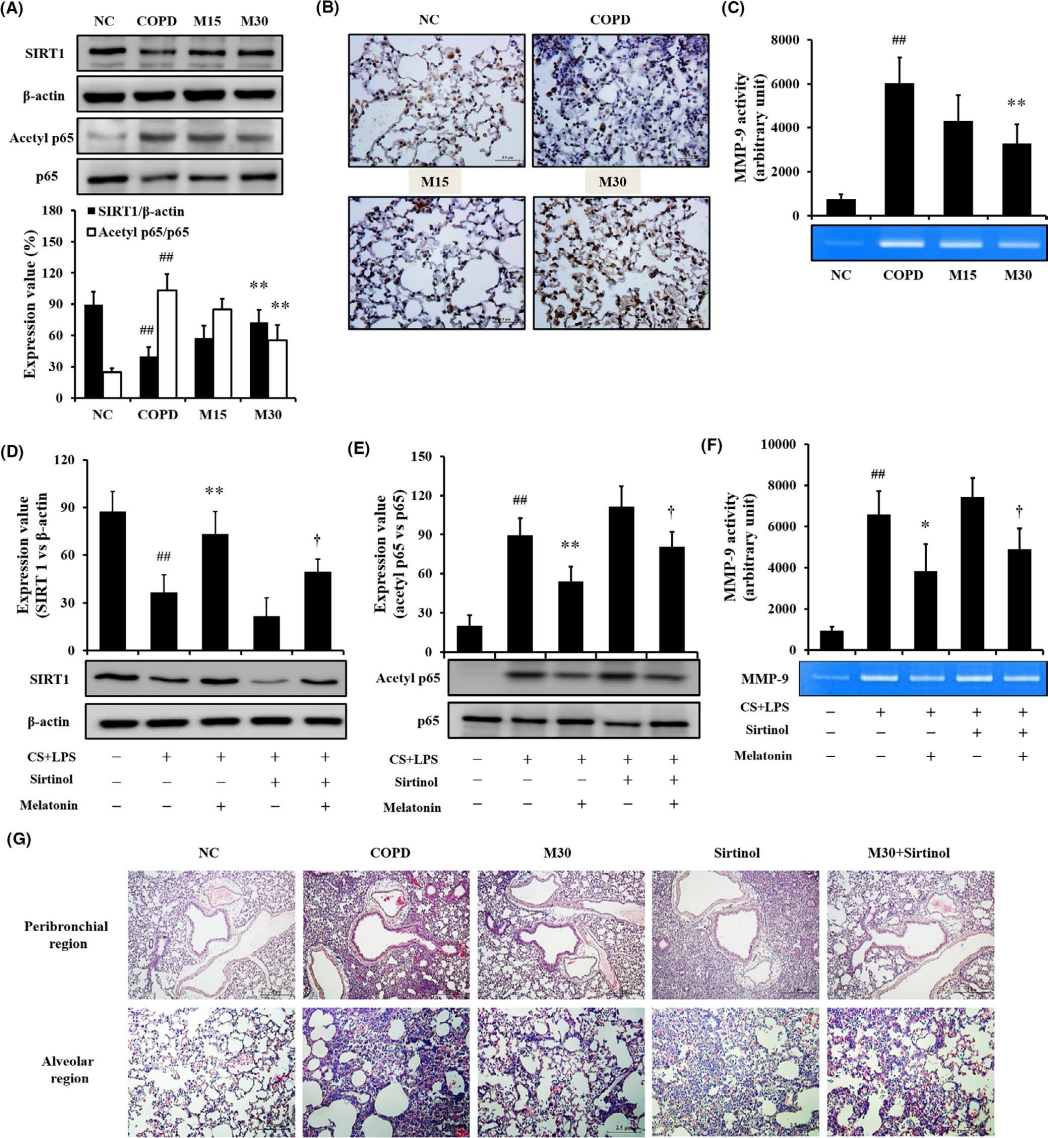
Melatonin suppressed the reduction in SIRT1 expression caused by cigarette smoke (CS) and lipopolysaccharide (LPS). A, SIRT1 expression on the gel; B, SIRT1 expression in the lung tissue. C, MMP‐9 activity; D, SIRT1 expression on the gel in secondary in vivo experiment; E, p65 acetylation in secondary in vivo experiment; F, MMP‐9 activity in secondary in vivo experiment; G, Histology of lung tissue in secondary in vivo experiment. To evaluate the effect of melatonin on SIRT1 expression, immunohistochemistry and Western blotting were performed. NC: normal control mice; COPD: CS + LPS‐exposed mice; Mel 15 and 30, CS + LPS‐exposed mice treated with melatonin (15 and 30 mg/kg, respectively), Sirtinol, CS + LPS‐exposed mice treated with sirtinol (10 mg/kg), M30 + Sirtinol, CS + LPS‐exposed mice treated with sirtinol (10 mg/kg) and melatonin (30 mg/kg). ^##^ *P* < .01, compared with the normal control mice; **P < .01, compared with the CS + LPS‐exposed mice; **^†^** *P* < .05, compared with CS + LPS‐exposed mice treated with sirtinol. The experimental animals were used five mice per group. The data are presented as mean ± standard deviation (SD). Statistical analyses were performed using analysis of variance, followed by a multiple comparison test with Bonferroni adjustment using GraphPad Prism 6

Treatment of melatonin in COPD mice decreased inflammatory mediators in BALF and inflammatory cell infiltration into lung tissue (Figures S3 and S4). Treatment of melatonin in COPD mice increased SIRT1 expression and decreased p65 acetylation (Figure 2A,B) with the reduction in MMP‐9 activity (Figure 2C).

### 3.3 Effect of melatonin on regulation of SIRT1 expression in mice exposed to CS

The CS‐exposed mice treated with sirtinol exhibited an increase in the inflammatory mediators compared with that of COPD mice (Figure S5). In contrast, cotreatment of COPD mice with sirtinol and melatonin reduced the inflammatory mediators compared with those of COPD mice treated with sirtinol alone. COPD mice treated with sirtinol exhibited lower SIRT1 expression than that in COPD mice; however, SIRT1 expression was restored by melatonin treatment (Figure 2D). The acetylation of p65 was increased in CS‐ and LPS‐exposed mice treated with sirtinol compared with that of CS‐ and LPS‐exposed mice; however, the increase in acetylation was reduced by melatonin treatment (Figure 2E) with the reduction in MMP‐9 activity (Figure 2F). Histological examination was similar to the results of Western blot (Figure 2G).

## 4 DISCUSSION

During the development of COPD, inflammatory cells act as initiators and accelerants in the airway inflammatory responses, because they produce various cytokines, chemokines, reactive oxygen species and proteases, which result in the inflammation of the airway and the destruction of normal alveolar structures.^11^ In this study, melatonin effectively inhibited the inflammatory mediators produced by CSC or CS exposure in both in vitro and in vivo experiments. The pharmacological effects of melatonin were accompanied by an increase in SIRT1 expression and a decrease in p65 acetylation. These findings suggested that the therapeutic properties of melatonin against CS‐ or CSC‐induced inflammation were closely associated with the regulation of SIRT1/p65 signalling.

SIRT1 is an NAD‐dependent histone deacetylation enzyme that is associated with various cellular processes via the regulation of activity of various transcription factors, including NF‐κB.^12^ In the inflammatory process, the overexpression of SIRT1 induces an anti‐inflammatory response via the inhibition of acetylation of transcription factors.^12^ During the development of COPD, SIRT1 expression was markedly decreased in CS‐treated human and model animals.^4^, ^13^ This reduction in SIRT1 expression eventually induces the inflammatory response via increase in the acetylation of transcription factors such as NF‐κB and FOXO3. In contrast, the enhanced expression of SIRT1 reduces the pathophysiological alterations induced by CS exposure.^14^ In the present study, melatonin treatment, which enhanced SIRT1 expression in untreated J774 macrophages, restored the reduction in SIRT1 expression caused by CSC treatment with the decrease in p65 acetylation in in vivo and in vitro experiment. These results were supported by the histological analysis results. These results indicated that the therapeutic effects of melatonin on CS‐caused airway inflammation are closely related to the enhancement of SIRT1 expression. In secondary in vivo experiments using an SIRT1 inhibitor, treatment of CS‐exposed mice with sirtinol decreased SIRT1 expression and increased p65 acetylation compared with those in CS‐treated mice, which was accompanied by an elevation in inflammatory mediators, including inflammatory cells, IL‐6, TNF‐α and MMP‐9. In contrast, the cotreatment of mice with melatonin and sirtinol increased SIRT1 expression compared with that of sirtinol‐treated mice, which induced a reduction in the acetylation of p65, resulting in the decreased production of inflammatory mediators. These results indicated that melatonin enhanced SIRT1 expression in the lung tissue, reducing pathophysiological alterations induced by CS.

In conclusion, melatonin reduced the inflammatory mediators in CS‐exposed mice and CSC‐treated J774 macrophages, which were closely related to enhanced SIRT1 expression induced by melatonin. Therefore, our results suggested that melatonin, an SIRT1 enhancer, may be an effective therapeutic agent to control CS‐induced airway inflammation.

## CONFLICT OF INTEREST

The authors declare that they have no conflict of interest.

## AUTHOR CONTRIBUTIONS

ISS and JSK conceived and directed the project, including experimental design and data interpretation. NRS, JWK and GP contributed to the experimental design and performed all of in vivo and in vitro experiments. NRS and JCK prepared the manuscript.

## Data Availability

The data that support the finding of this study are available from the corresponding author upon reasonable request.
