# Supplementary material for: Role of melatonin as an SIRT1 enhancer in chronic obstructive pulmonary disease induced by cigarette smoke
Source: J Cell Mol Med. 2019 Nov 25;24(1):1151–6. doi: 10.1111/jcmm.14816 (PMC6933319; doi:10.1111/jcmm.14816)
Supplement: Supplementary file 1 [file JCMM-24-1151-s001.docx]

**Supplementary Figure**

**
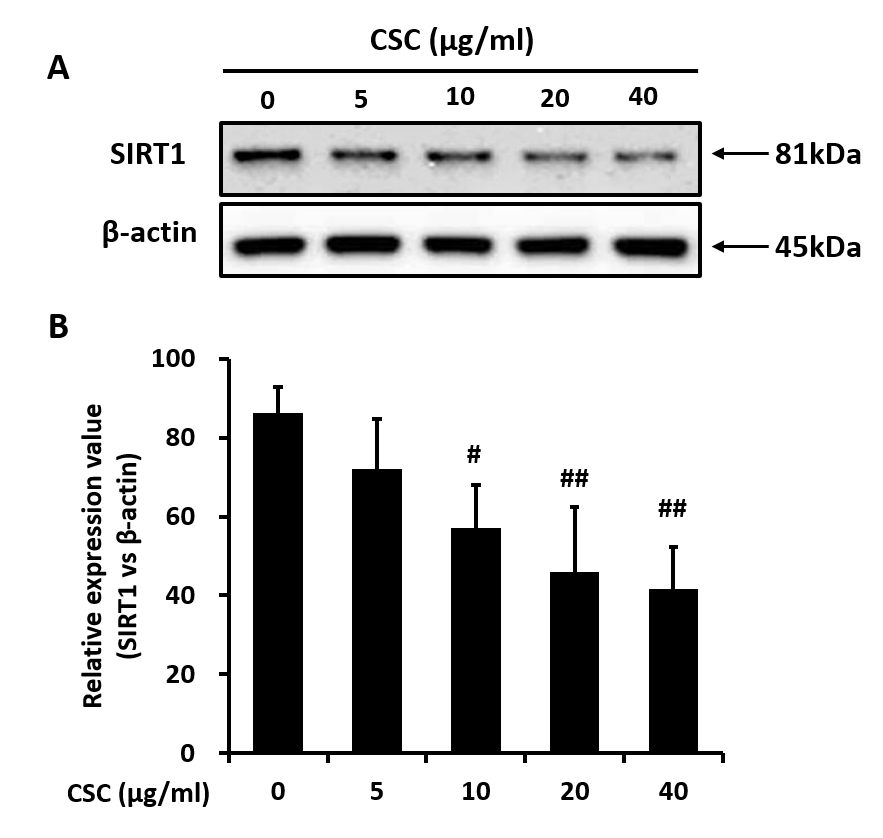
**

**Figure S1** Cigarette smoke condensate (CSC) decreases SIRT1 expression in concentration dependent manner. (A) SIRT1 expression on gel, (B) Relative expression value (SIRT1 vs β-actin). ^#^,^##^Significantly different from non-stimulated J774 macrophage cells, P < 0.05 and 0.01, respectively. The data are presented as mean ± standard deviation (SD). Statistical analyses were performed using analysis of variance, followed by a multiple comparison test with Dunnett adjustment using GraphPad Prism 6.

**Figure S2** Melatonin suppresses the production of TNF-α in cigarette smoke condensate (CSC) stimulated J774 macrophage cells. The level of TNF-α was determined using commercial ELIS kit. ^*^,^**^Significantly different from CSC stimulated J774 macrophage cells, P < 0.05 and 0.01, respectively. The data are presented as mean ± standard deviation (SD). Statistical analyses were performed using analysis of variance, followed by a multiple comparison test with Dunnett adjustment using GraphPad Prism 6.


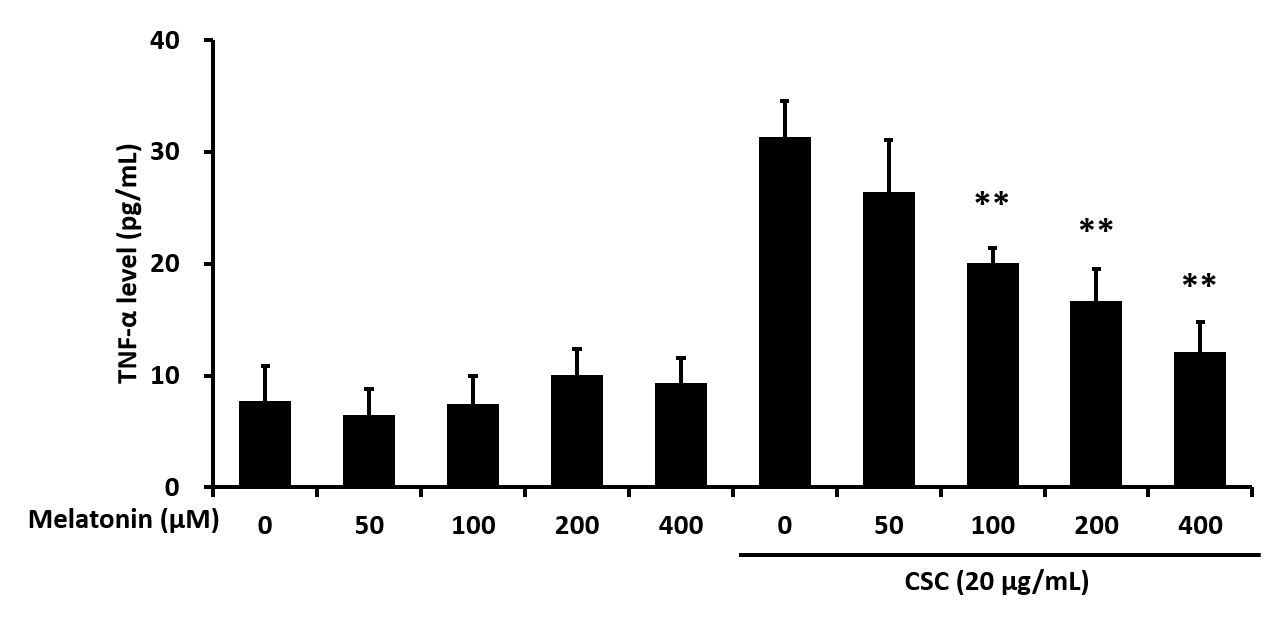


**Figure S3** Melatonin decreases inflammatory cell count, IL-6, TNF-α and BLAF protein in bronchoalveolar lavage fluids of cigarette smoke (CS) and lipopolysaccharide (LPS) exposed animals. (A) Neutrophils counts, (B) total cell counts, (C) IL-6 level, (D), TNF-α level, (E) BALF protein. NC: normal control animals; COPD: CS + LPS intranasal instillation induced mice; Mel 15 and 30, melatonin (15 mg/kg and 30 mg/kg, respectively) + CS + LPS intranasal instillation. ^##^Significantly different from the NC, *P* < 0.01; ^*^,^**^Significantly different from the COPD, *P* < 0.05 and 0.01, respectively. The data are presented as mean ± standard deviation (SD). Statistical analyses were performed using analysis of variance, followed by a multiple comparison test with Dunnett adjustment using GraphPad Prism 6.


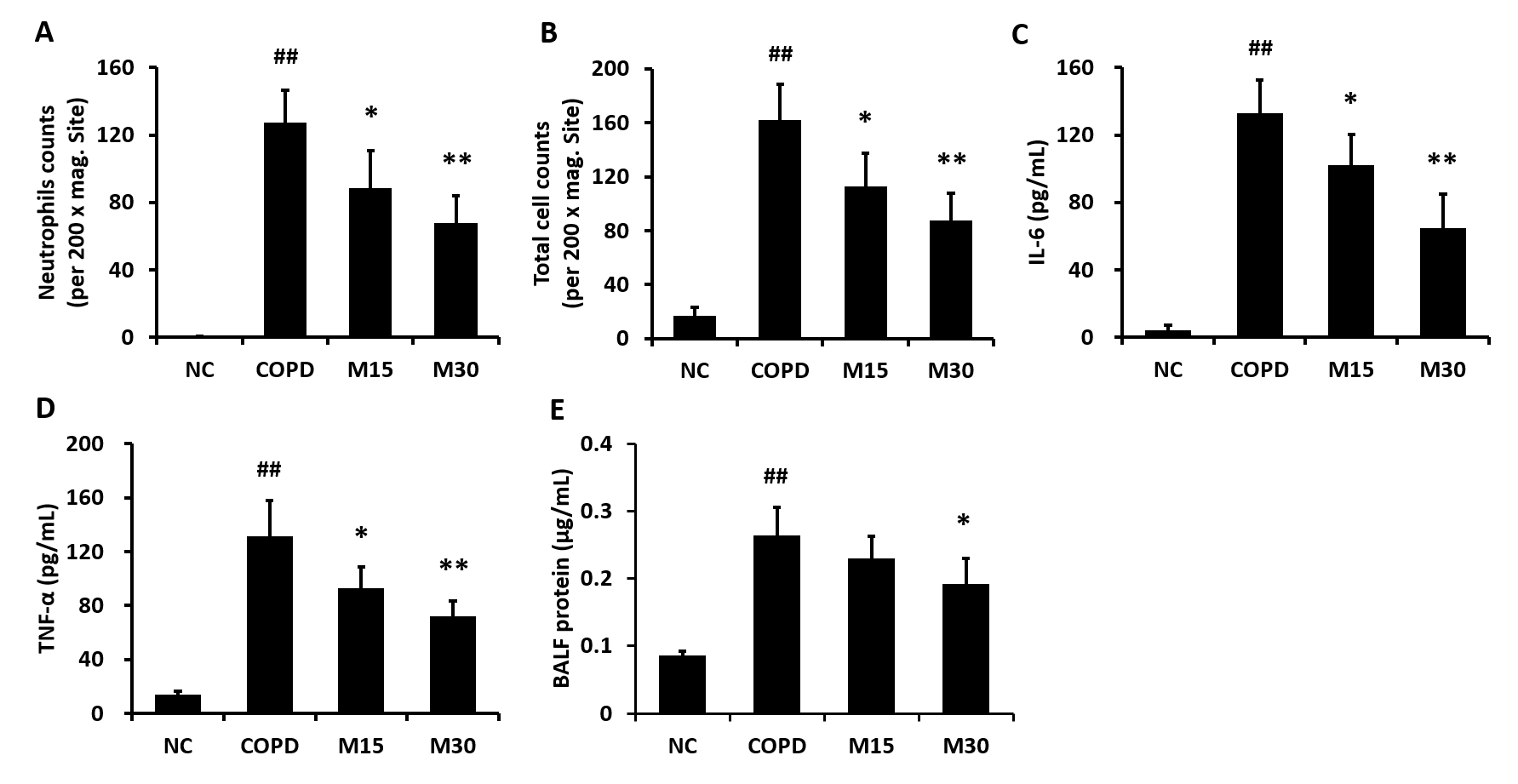


**Figure S4** Melatonin reduces inflammatory responses in cigarette smoke (CS) and lipopolysaccharide (LPS) exposed animals. NC: normal control animals; COPD: CS + LPS intranasal instillation induced mice; Mel 15 and 30, melatonin (15 mg/kg and 30 mg/kg, respectively) + CS + LPS intranasal instillation.


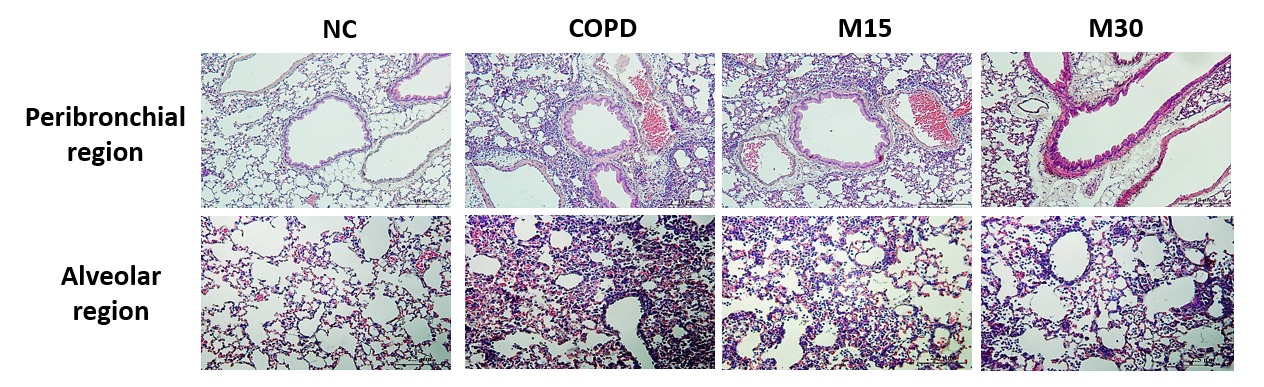


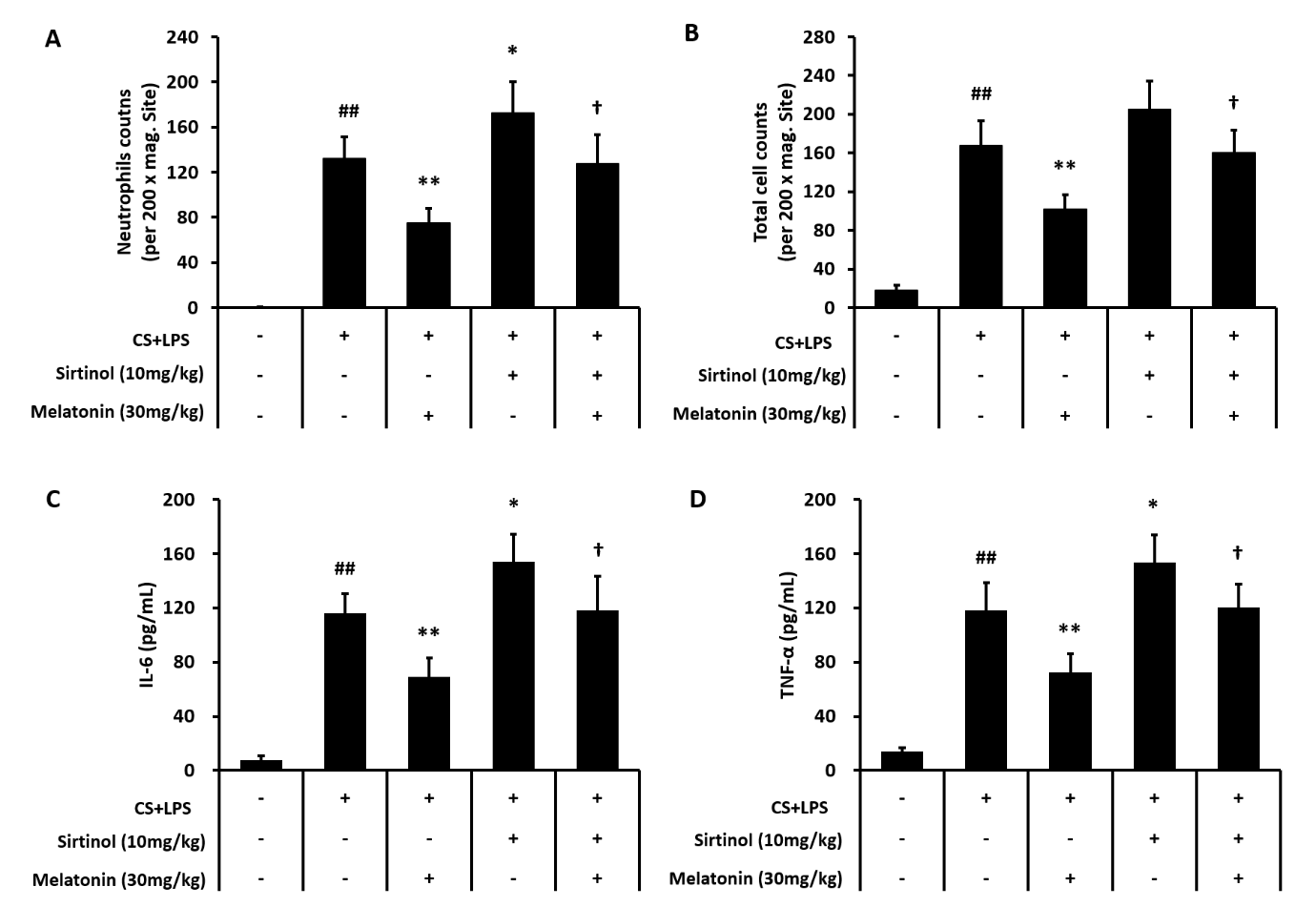


**Figure S5** Melatonin decreased the inflammatory cell count, and IL-6 and TNF-α levels in bronchoalveolar lavage fluid of mice induced by cigarette smoke (CS) and lipopolysaccharide (LPS). (A) Neutrophil count; (B) total cell count; (C) IL-6 level; (D) TNF-α level. ^##^*P* < 0.01, compared with the normal control mice; ^*^*P* < 0.05 and ^**^*P* < 0.01 compared with the CS + LPS-exposed mice; **^†^***P* < 0.05, compared with the CS + LPS-exposed mice treated with sirtinol. The experimental animals were used 5 mouse per group. The data are presented as mean ± standard deviation (SD). Statistical analyses were performed using analysis of variance, followed by a multiple comparison test with Bonferroni adjustment using GraphPad Prism 6.
